# The INDUS knee prosthesis – Prospective multicentric trial of a posteriorly stabilized high-flex design: 2 years follow-up

**DOI:** 10.4103/0019-5413.55976

**Published:** 2009

**Authors:** Kantilal H Sancheti, Nandu S Laud, Harish Bhende, Gurava Reddy, Neema Pramod, Joseph N Mani

**Affiliations:** Sancheti Institute for Orthopaedics and Rehabilitation, 16, Shivajinagar, Pune - 411 005, India; 1 Laud Clinic, 180, Saraswati Neelayam Colony, Dadar (East), Mumbai - 400 014, India; 2 Sumshine, Secundrabad - 500 003, India; 3 Unique Super Speciality Centre, 714/715, Vijay Syndicate, Annapurna Road, Indore - 59, India; 4 Department of Orthopaedics, Baby Memorial Hospital, Calicut - 673 004, India

**Keywords:** Backside wear, high flexion, INDUS knee

## Abstract

**Background:**

The anatomical and morphological differences and high-flexion daily activities in the Asian population have since ever prompted for development of customized knee replacement systems. INDUS knee system has advantages both of high-flex designs and is developed by keeping the anatomical variations of the native population in mind. The purpose of this study is to analyze the 2-year follow-up results using the INDUS prosthesis.

**Materials and Methods:**

Two hundred and ninety-seven knees in 276 patients were prospectively analyzed. There were 65 men (72 knees) and 211 (225 knees) women with a mean age of 64.56 years. Two hundred and forty-five knees had primary osteoarthritis, 48 knees had rheumatoid arthritis, and four knees had post-traumatic arthritis. Clinical parameters, including the Knee Society scores (knee score and function score), range of motion, post-operative anterior knee pain, and complications were recorded. Pre- and post-operative serial radiographs were analyzed for limb alignment, component positioning, and evidence of loosening.

**Results:**

The patients were followed-up for an average of 2.59 years (range, 2–3.3 years). The mean knee score and the mean function score were significantly improved from a pre-operative value of 39.4 points and 46.7 points to a post-operative value of 87 points and 86 points, respectively (*P* value <0.05). Two hundred and thirty four knees had no anterior knee pain while 63 knees had mild to moderate pain, but none of the patients requested any intervention for the same. Of the 276 patients (297 knees), 79 knees had flexion above 140°, 167 had a flexion range of 130–140°, 27 had a flexion range of 100–130°, and 24 knees had a flexion < 100°, with the mean range of movement being 132.9°. Improvements in the range of movement were retained over time and a total of 205 patients (224 knees, 75.7%) could squat or sit cross-legged at the final follow-up. The mean tibiofemoral angle was 8.5°±6.9° of varus pre-operatively and 5.4°±2.2° of valgus (3–7° of valgus) at the final follow-up, with no loss of alignment noted in any case. One knee underwent revision for late infection while another knee had periprosthetic supracondylar fracture treated with plate fixation.

**Conclusions:**

Use of the INDUS knee prosthesis has a favorable short-term outcome, with a mean range of 135° flexion and excellent knee scores.

## INTRODUCTION

Total joint replacement is the technologically advanced solution for arthritic pain. However, a search for a better functional and durable prosthesis still continues. The original total condylar design was very successful in terms of pain relief and durability, but the average post-operative flexion achieved was only around 90–95°.^1^–^7^ Even though this may be enough for most of the daily activities in the western world,^8^ Asians, and particularly Indians, require a higher flexion for most of their daily social habits and customs.^9^ In 1978, the posterior stabilized condylar prosthesis was introduced, as a modification of the total condylar prosthesis, by Insall *et al*.^10^ In this prosthesis, a post and cam mechanism was used to achieve femoral rollback. The average flexion achieved by this prosthesis was 107–115°.^10^–^14^ Similarly, cruciate retaining designs achieve a flexion of around 110–112°.^15^,^16^ Although this was a significant improvement, it was not enough for daily habits like cross-legged sitting and squatting that are so common in the Indian subcontinent. In recent times, a number of additional design modifications have been introduced to achieve this goal.^17^,^18^ This increase in flexion however brings with it the fear of loosening and excessive wear.^19^ Moreover, most modern knee designs are modular, which results in backside wear.^20^ Current design trends are focusing on shortening the radii of curvature; such shortening, in turn, thickens the posterior femoral condyle and increases the height of the posterior-stabilized box, both of which require removal of more bone. The end results may be excessive wear, increased patellofemoral complications, and difficult revisions.

The INDUS prosthesis is designed so as to keep the thickness of the posterior condyle equivalent to the thickness of the distal condyle. Also, the cam and post have been placed in such a way that the removal of the box in the center is much less; hence, patellofemoral complications are much lower.

The purpose of this study is to analyze the 2 year follow-up results using the INDUS prosthesis. This prosthesis has been in use at six centers since August 2005.

## MATERIALS AND METHODS

This study was approved by the institutional ethical committee of the hospital. The implant design was approved and funded by Division of Science and Technology (DST). Three hundred and seventy-eight primary total knee arthroplasties in 343 patients using the INDUS knee prosthesis were prospectively enrolled in this study that was conducted at six centers. The surgeries were performed from August 2005 to October 2006. Three patients died (four knees) in the first year of surgery due to unrelated causes. Seventeen patients were lost to follow-up (23 knees). For the purpose of reporting the short-term results, we excluded 47 patients with less than 2 years of follow-up (54 knees). Thus, a total of 297 knees (276 patients) were available for review. A demographic detail of patients from different centers is shown in Table 1. There were 65 men (72 knees) and 211 (225 knees) women. The mean age was 64.56 years (49–91). Two hundred and fifty-five patients underwent unilateral total knee replacement (TKR) and 21 patients underwent bilateral TKR at an average interval of 2.4 months (range, 1–3 months). The diagnosis was primary osteoarthritis in 245 knees (82.49%, 14 bilateral), rheumatoid arthritis in 48 knees (16.16%, seven bilateral), and post-traumatic arthritis in four (1.34%) knees. The patients were followed-up for an average of 2.59 years (range, 2–3.3 years).

**Table 1:** Demographic details from different centers

Variables	No. of patients	Mean age (years)	Male/female (no of knees)	Mean BMI	Mean follow-up (years)
Center 1	166 (178 knees)	64.7	27/139	28.5	3.16
Center 2	25 (31 knees)	61.32	4/21	27.4	2.65
Center 3	19 (19 knees)	66.26	6/13	26.3	2.58
Center 4	32 (34 knees)	66.5	13/19	23.9	2.56
Center 5	23 (24 knees)	62.74	11/12	27.6	2.56
Center 6	11 (11 knees)	64.91	4/7	24.1	2.5
Total	276 (297 knees)	64.56	72/225	26.3	2.66

The mean body mass index from all the centers was 26.5.

### The implant

**Figure 1:**
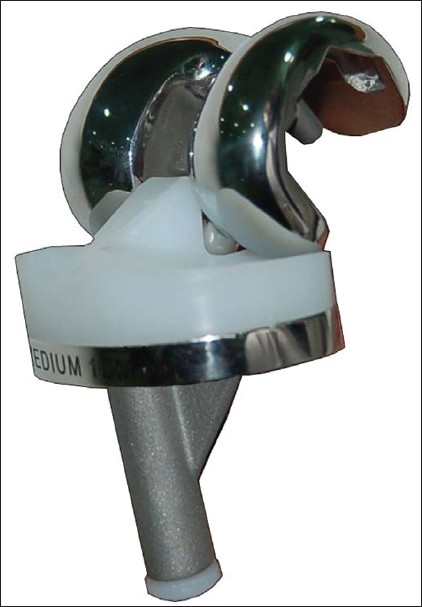
INDUS a posterior stabilized design to increase femoral rollback

**Figure 2:**
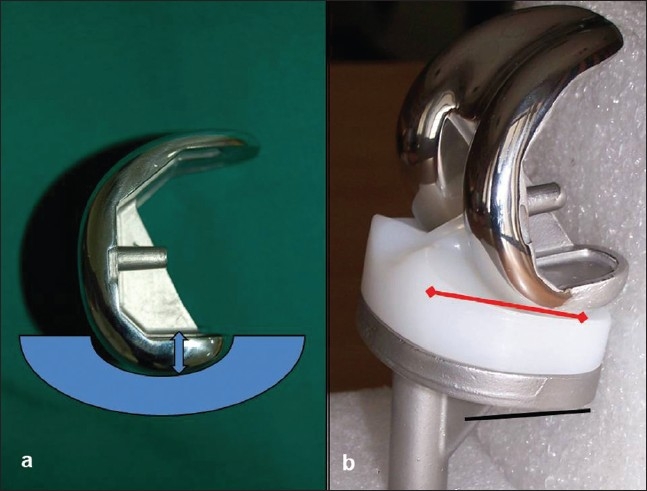
INDUS Hiflex features. (a) An improved J curve (thicker posterior condyle) increases the posterior offset. (b) The tibial component having total slope of 70 with 40 in the base plate (black line) and 30 in the insert (red arrow)

**Figure 3:**
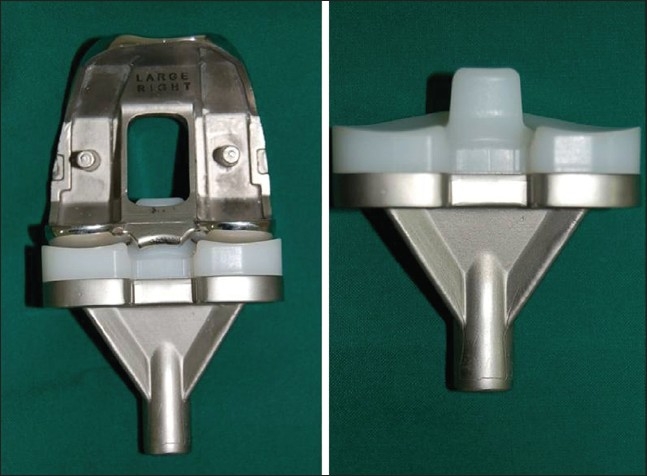
The post and cam conform mediolaterally to form a third joint allowing enhanced rotational congruency

**Figure 4:**
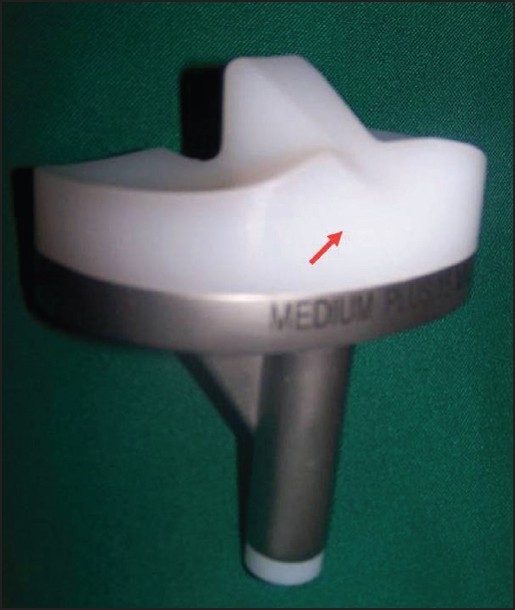
Monoblock tibial component with deep dish design and anterior cut out in insert (arrow) to accommodate the patellar tendon during deep knee flexion

**Figure 5:**
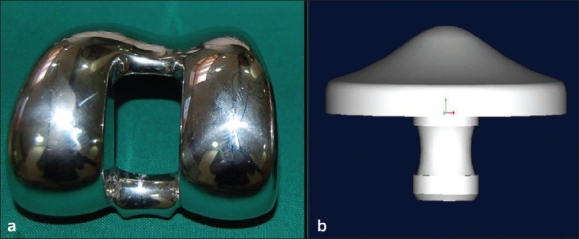
(a) Anatomical trochlear design with open intercondylar box. (b) single peg anatomical patella

The ethnic anatomical and cultural variations in the Indian population have prompted the need for development of the INDUS Knee.^21^ It is a posterior cruciate substituting design where femoral rollback occurs by a post and cam mechanism [Figure 1]. The radius of curvature of the posterior condyle of the femoral component (J curve) has been reduced, thereby increasing the posterior condylar offset. The total thickness remains the same. The original offset is reproduced. This helps in gaining more femoral rollback and more flexion. A 4° slope is incorporated in the tibial insert and a 3° slope in the metal base plate to aid in increase of flexion^22^ [Figure 2]. The above two design modifications allow maximal flexion at the knee. The deep flexion achieved prompted certain novel modifications in the post and cam mechanism to offer stability and to allow for rotational freedom in deep flexion. A third joint was designed between the post and the cam, with the post engaging the cam at around 80° of flexion and thereafter acting like a load bearing surface in deep flexion. The articulating surface of the post is convex and that of the cam is concave thus allowing a more congruent surface for allowing load transfer and the rotation to occur [Figure 3] as the post does not impinge on the side walls of the box during rotations. This rotational freedom is necessary to prevent wear of the post and hence loosening and osteolysis of the tibial component.^23^ Also, as the bar of the cam articulates with the post at a lower level, the jumping distance is increased to 16 mm, another feature to assist in enhanced stability. The posterior edges of the tibial polyethylene insert are chamfered to avoid impingement in deep knee flexion. There is an anterior cutout in the tibial polyethylene insert to accommodate the patellar tendon during deep flexion [Figure 4]. The tibia is monoblock and hence backside wear is reduced to a minimum.^20^ The tibial polyethylene insert has a deep dish design to prevent point loading and polywear [Figure 4]. Introduction of the post and cam mechanism involves removal of extra bone from the intercondylar region of the femur to accommodate the box. This results in bone loss.^10^,^13^,^24^ In the INDUS design, the femoral box is designed so as to cut minimal bone. Thus, INDUS knee also incorporates the bone sparing principle. A thorough anatomic study of the Indian knee joints was done before designing the components. The components are made in sizes that are more suited to the Indian population. The various sizes were designed in accordance with the suggestions of Vaidhya *et al*.^21^ and by analyses of 100 computerized tomography scans in 50 patients (independent unpublished data) The femoral components are separate for the right and left, with an anatomic deep trochlear design for better patellar tracking and avoiding the patellar clunk syndrome.^25^,^26^ The intercondylar box of the femoral component is kept open to enable nailing in case of periprosthetic fractures. The patella is a single peg anatomic design [Figure 5]. In conclusion, the INDUS knee is designed to be a high-flexion, bone sparing and anatomic design suitable for the native population. The prosthesis is Gamma irradiated in vacuum to increase the longevity of the polyethylene.^27^

### Operative procedure

Before the operation, full-length weight-bearing radiographs were obtained showing the hip, knee, and ankle joints. The angles of the femoral and tibial cuts and the desired position of the femoral entry hole were planned before surgery. An anterior midline incision measuring 12–14 cm was followed by a medial parapatellar arthrotomy. All the components were cemented after pulsed lavage and drying of the cut bone ends and pressurization of the cement. Patelloplasty rather than patellar resurfacing was performed in 111 knees when the articular cartilage was found to be healthy, erosion was <20% and when patellar tracking was good especially in younger patients. It consisted of a peripatellar synovectomy, electrocautery of the patellar rim to provide partial denervation, and removal of osteophytes. The remaining patellae were resurfaced. All patients received prophylactic antibiotics. Prophylactic antibiotics were given one night before and 45 min before surgery. Intravenous antibiotics were continued for 3 days after surgery. They wore compression stockings for 2 weeks. A drain was used for 24 h. Active quadriceps-strengthening exercises and range of motion were commenced immediately post-operatively and continuous passive motion (CPM) machine was not used. Weight-bearing was allowed from the second post-operative day. Knee flexion was allowed as guided by the pain tolerance of the patient.

The patients were reviewed at 6 weeks, 3 months, 6 months, 1 year, and annually thereafter. Clinical parameters, including the Knee Society scores (KSS),^28^ range of motion, post-operative clinical anterior knee pain and complications were recorded. The KSS was evaluated in two parts: first, the knee score involving the clinical knee findings, and, second, the function score analyzes functional mobility of the patient. In addition, patients were asked to squat or sit cross-legged at follow-up. Pre-operative factors affecting the post-operative range of movement were also analyzed, including flexion contracture, deformity, and range of movement. No subjective parameter was recorded.

Pre- and post-operative weight bearing radiographs included anteroposterior, lateral, and full-length anteroposterior scanogram films and a skyline patellar view. These were assessed for limb alignment, component positioning, and the presence and location of radiolucent lines at the bonecement interface.

Statistical comparisons of the pre-operative and post-operative clinical scores (categorical variable) were made with the use of chi-square analysis. Correlations between the pre-operative and post-operative ranges of flexion (continuous variable) were investigated with the paired t-test. The level of statistically significant difference was set at *P* = 0.05.

## RESULTS

**Figure 6:**
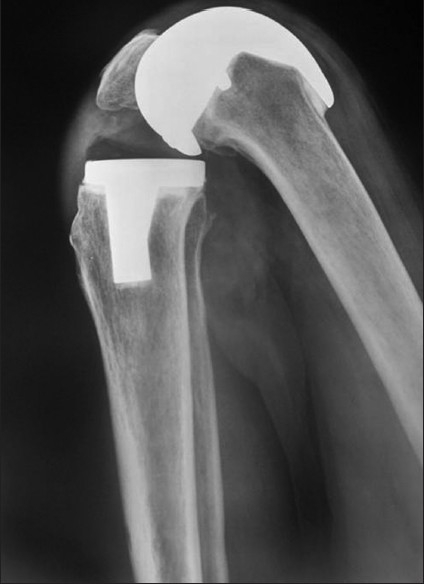
Lateral radiograph of the knee of three year follow up of INDUS knee showing maintaining optimal flexion

The patients were followed-up for an average of 2.59 years (2–3.3 years). A change in values of different variables from all the six centers is shown in Table 2. Pre-operatively, the mean knee score was 39.4 ± 11.4 points and the mean function score was 46.7 ± 17.4 points. Both scores showed significant improvement at final follow-up [Table 3]. At the last follow-up, the clinical anterior knee pain scores were grade 0 (no pain) in 234 knees (78.79%), grade 1 (mild pain) in 35 knees (11.78%), and grade 2 (moderate pain) in 28 knees (9.43%); none were grade 3 (severe pain). None of the patients requested a revision because of anterior knee pain. A total of 205 patients (224 knees, 75.7%) could squat or sit cross-legged at the final follow-up. Pre-operatively, the mean flexion contracture was 10.3° (range, 0–30°), the mean maximum flexion was 114.83° (60–140°), and the mean range of movement was 106.2° (45–150°). At the last follow-up, the mean flexion contracture was 3.19° (0–10°), the mean maximal flexion angle was 136.14° (90–160°), and the mean range of movement was 132.10° (85–160°). These outcomes represent a statistically significant improvement [Table 3]. Of the 276 patients (297 knees), 79 (26.6%) knees had flexion above 140°, 167 (56.23%) had a flexion range of 130–140°, 27 (9.09%) had a flexion range of 100–130° and 24 (8.08%) knees had flexion <100°. Improvements in the range of movement were retained over time [Figure 6]. No clunks or crepitus were heard.

**Table 2:** Pre- and post-operative data of all the centers

Variables	Flexion deformity (degrees)		Range of motion (degrees)		Total knee score		Total functional score	
Variables								
	Pre-op	Post-op	Pre-op	Post-op	Pre-op	Post-op	Pre-op	Post-op
Center 1	11.01	3.37	102.67	133	38.39	93.9	43.89	91.43
Center 2	10.48	2.58	101.13	134.84	39.77	93.16	46.61	87.45
Center 3	8.95	1.84	107.89	133.42	38.79	84.68	47.74	84.47
Centre 4	12.06	3.09	103.82	133.24	40.5	84.71	49.65	84.41
Center 5	11.88	4.17	108.54	131.25	38.25	85.25	49.04	84.79
Center 6	10	4.09	113.64	127.27	41.18	83.82	43.55	83.73
Total	10.73	3.19	106.28	132.17	39.48	87.58	46.74	86.04

**Table 3:** Comparison of pre-operative and post-operative clinical variables

Variable	Pre-operative (mean ± SD)	Post-operative (mean ± SD)	*P* value
Knee score*	39.4 ± 11.4	87.5 ± 7.16	<0.05
Function score*	46.7 ± 17.4	86 ± 9.30	<0.05
Flexion deformity#	10.7°	3.19°	0.041
Terminal flexion#	117.9°	135.4°	0.028
Range of motion#	106.2°	132.1°	0.034

* *P* values are for chi-square test,

# *P* values are for paired t-test

**Figure 7:**
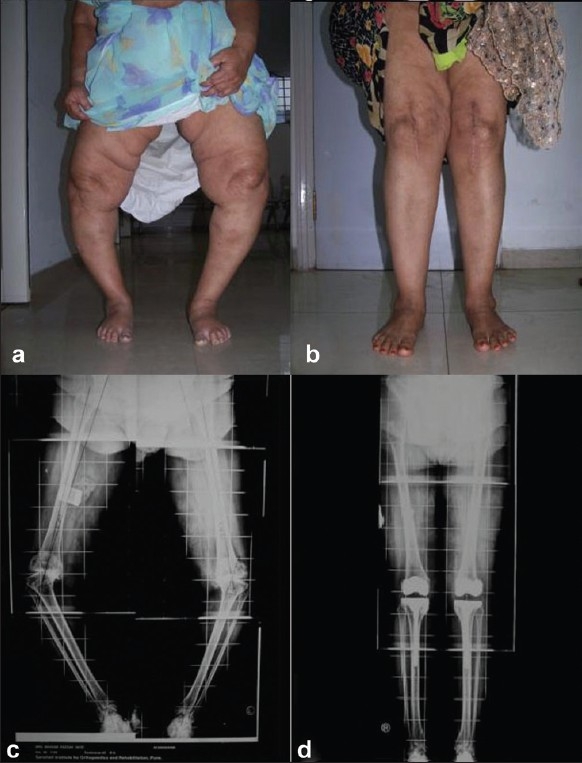
Clinical pre-operative photograph (a) of a 62 yrs old patient with severe genuvarum with OA. Clinical photograph (b) of same patient 30 months follow-up shows good deformity correction. Pre-operative scanogram (c) and post-operative scanogram (d) of the same patient shows good correction

**Figure 8:**
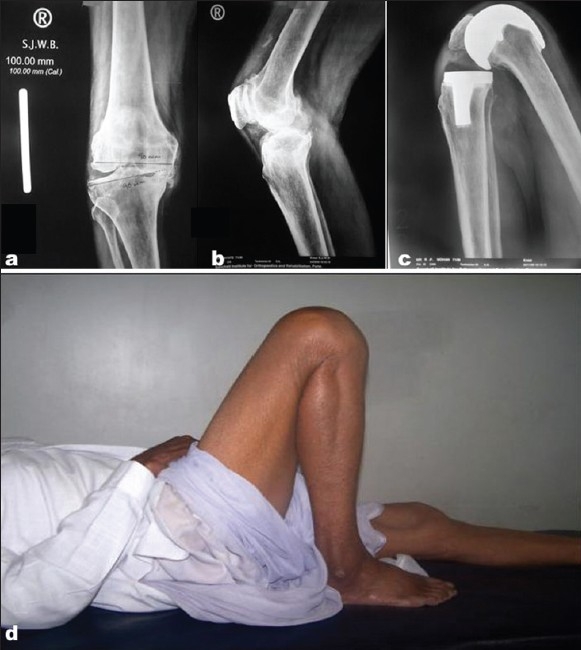
Pre-operative radiographs antero-posterior view (a) and lateral view (b) of the knee of 71 years old patient shows severe OA with genu varum. Post-operative lateral radiograph (c) show range of flexion achieved and clinical photographs (d) shows the range of flexion at 28 months follow-up

A complete set of radiographs of 285 knees out of 297 were available for analysis at final follow-up [Figures 7 and 8].

The mean tibiofemoral angle was 8.5° ± 6.9° of varus (32° of varus to 18° of valgus) pre-operatively and 5.4° ± 2.2° of valgus (3°–7° of valgus) at final follow-up. No change in alignment was observed at any post-operative radiological examination. Radiolucent lines were observed in 58 knees. All were seen below the tibial base plate beginning at around 1 year of follow-up and were non-progressive. There was no evidence of loosening of the tibial component. There were no cases of femoral radiolucency.

One knee underwent a two-stage revision because of late infection with *Staphylococcus aureus*, which was identified 7 months after surgery. In another knee, a peri-prosthetic supracondylar fracture of the femur occurred 17 months after operation, and this was treated successfully with open reduction and plate fixation. The femoral component remained well fixed.

## DISCUSSION

Achieving maximal knee flexion improves the patient satisfaction and knee performance. However, conventional knee arthroplasty before high-flex designs rarely allows for knee flexion beyond 120°.^29^,^30^ The newer high-flexion knee replacement prostheses have reported a higher flexion achieved post-operatively, within a range of 130–135° of flexion.^31^,^34^ The INDUS knee is an indigineously researched and designed knee prosthesis that achieves a mean flexion of 135° without compromising on stability and polyethylene wear and with minimal bone resection.

Component sizing and proper placement of the components is key to the success of knee arthroplasty.^35^,^36^ Component oversizing in the anteroposterior dimension alters the delicate balance in flexion extension gap, resulting in flexion tightness post-operatively because of increased tension in the quadriceps mechanism^36^ whereas oversizing in the mediolateral dimension affects the patellar tracking and wound closure.^37^ The risk of component oversizing with the other available implants is especially present in Indians and other Asian subpopulations that are known to have a smaller build and stature as compared with the western population.^21^,^38^,^39^ INDUS knee is based on careful assessment of anthropometric data from the Indian patients thus reducing the risk of oversizing.

A number of modifications have been made in the post and cam mechanism of the INDUS knee to enhance flexion and increase stability in deep flexion. Han *et al*.^40^ reported a high incidence of loosening of the femoral component in a retrospective series of 72 knees using the Legacy posterior stabilized–flex total knee replacement. At a mean follow-up of 32 months, aseptic loosening of the femoral component was found in 27 (38%) cases, with 15 (21%) requiring revision at a mean of 23 months. They concluded that the high rate of loosening was associated with weight bearing at maximum flexion and inadequate support of the posterior condyle of the prosthesis. However, in the INDUS knee, the modified post and cam functions as a load-bearing third joint and lend support to the posterior condyles during deep flexion. Our follow-up is short, but we still have had no cases of mechanical loosening of either of the components.

Lack of rotational freedom of the post in the femoral box has been cited as a reason for causing polywear and osteolysis.^23^ In INDUS, the post does not impinge on the side walls of the box during rotations hence addressing this issue.

Most modern implants are modular. However, with modularity comes the problem of backside wear, which often occurs in amounts enough to cause osteolysis and loosening.^20^ To avoid these problems, a monoblock design was preferred for the INDUS knee.

Introduction of the post and cam mechanism involves removal of extra bone from the intercondylar region of the femur to accommodate the box. This results in bone loss.^10^,^13^,^24^ In the INDUS design, modifications in the post and cam mechanism allow for less bone removal thus preserving bone stock.

For conventional implants, the range of movement following TKRs is reported to be approximately 110°^10^–^16^ and few patients can flex beyond 120°.^41^–^43^ The excellent range of movement observed in this study is consistent with the kinematic advantages of high-flexion implants demonstrated in several biomechanical studies.^17^,^18^,^31^–^34^

The introduction of high-flexion prosthesis has raised concerns that the increased flexion which enables patients to squat or sit cross-legged may compromise the long-term results.^19^ There is also concern regarding loosening of the implant caused by design modifications requiring removal of more bone from the posterior femoral condyles and the intercondylar area.^19^,^44^ However, our study shows favorable results, with mean values of 135° flexion and excellent total and function KSS. No revisions for aseptic loosening were required. There were no incidents of instability or dislocation in the deep flexion. No patient requested revision because of anterior knee pain on deep flexion.

The limitations of this study include a limited number of patients with a short follow-up. However, the prospective nature of our study allows us to follow these patients and we should be able to identify and correct any problems seen with our new design. We have more than 95% follow-up and we will report on medium and longer term results as they become available.

In the present series, total knee arthroplasty with the INDUS knee prosthesis resulted in an excellent range of motion and good functional outcome, and the durability of the prosthesis is promising.
